# Medtronic’s Hugo^™^ robotic surgery system for robot-assisted radical prostatectomy: a systematic review of current worldwide experiences

**DOI:** 10.1007/s11701-024-02113-3

**Published:** 2024-09-28

**Authors:** Mehrshad Sultani Tehrani, Andrew Shepherd, Ben Challacombe

**Affiliations:** 1 King’s College London, London, UK; 2 Guy’s and St Thomas’ NHS Foundation Trust, London, UK; 3 Adelaide Medical School, University of Adelaide, Adelaide, Australia

**Keywords:** RAS, RARP, Hugo, Da Vinci, Robotic-assisted radical prostatectomy

## Abstract

Urology’s pioneering role in surgical innovations, from cystoscopy to laparoscopic surgery, culminated in the twenty-first-century advent of robotic surgery. The dominant da Vinci^®^ system faced new competition following its 2019 patent expiration. Medtronic’s Hugo^™^ system emerged. Its growing global adoption, especially in robot-assisted radical prostatectomy (RARP), necessitates a systematic review, evaluating safety, feasibility, and comparison with established systems. A comprehensive search identified eligible studies of the Hugo^™^ robotic platform for RARP, presenting their current experiences. Following systematic screening, quality of eligible studies was assessed using ROBINS-I. Results then underwent a narrative synthesis. This systematic review analysed 19 eligible studies, consisting of 9 comparative and 10 single arm studies. Due to the non-randomised nature of the studies, a moderate risk of bias was concluded in most. On account of the high heterogeneity between studies, a narrative synthesis of data was enacted; categorised into themes relating to operative timings, transfer of skills, patient demographics, plus safety and feasibility. Eligible studies demonstrated the promise of the Hugo^™^ platform within these themes, in comparison to currently available platforms. Despite a paucity of high-quality randomised controlled trials, available evidence indicates Hugo^™^ as a promising, safe alternative for RARP. Positive experiences across diverse centres and surgeons revealed minimal differences in surgical outcomes compared to the established da Vinci^®^ system, fostering global Hugo^™^ adoption. Despite evidence demonstrating Hugo^™^ safety and comparability, the review underscores the scarcity of high-quality evidence, attributing it to early stage implementation challenges.

## Introduction

The field of urology has been at the forefront of surgical innovations, adapting novel techniques and technologies to benefit patient outcomes. This is well illustrated by historical milestones such as the use of cystoscopy in the late nineteenth century [1], emergence of laparoscopic surgery in the early twentieth century [2], and most recently, the advent of robotic surgery in the twenty-first century.

Since U.S. Food and Drug Administration (FDA) approval in 2000, the da Vinci^®^ surgical system developed by Intuitive Surgical, has ushered in a new age of minimally invasive surgery; with its robotic-assisted surgical platform boasting superior dexterity and precision as well as visualisation compared to prior laparoscopic techniques [3]. This system has dominated the robotic landscape due to its high-quality and long-standing intellectual patents [4]. However, the expiration of these patents in 2019 has allowed new competitors to emerge.

**Fig. 1:**
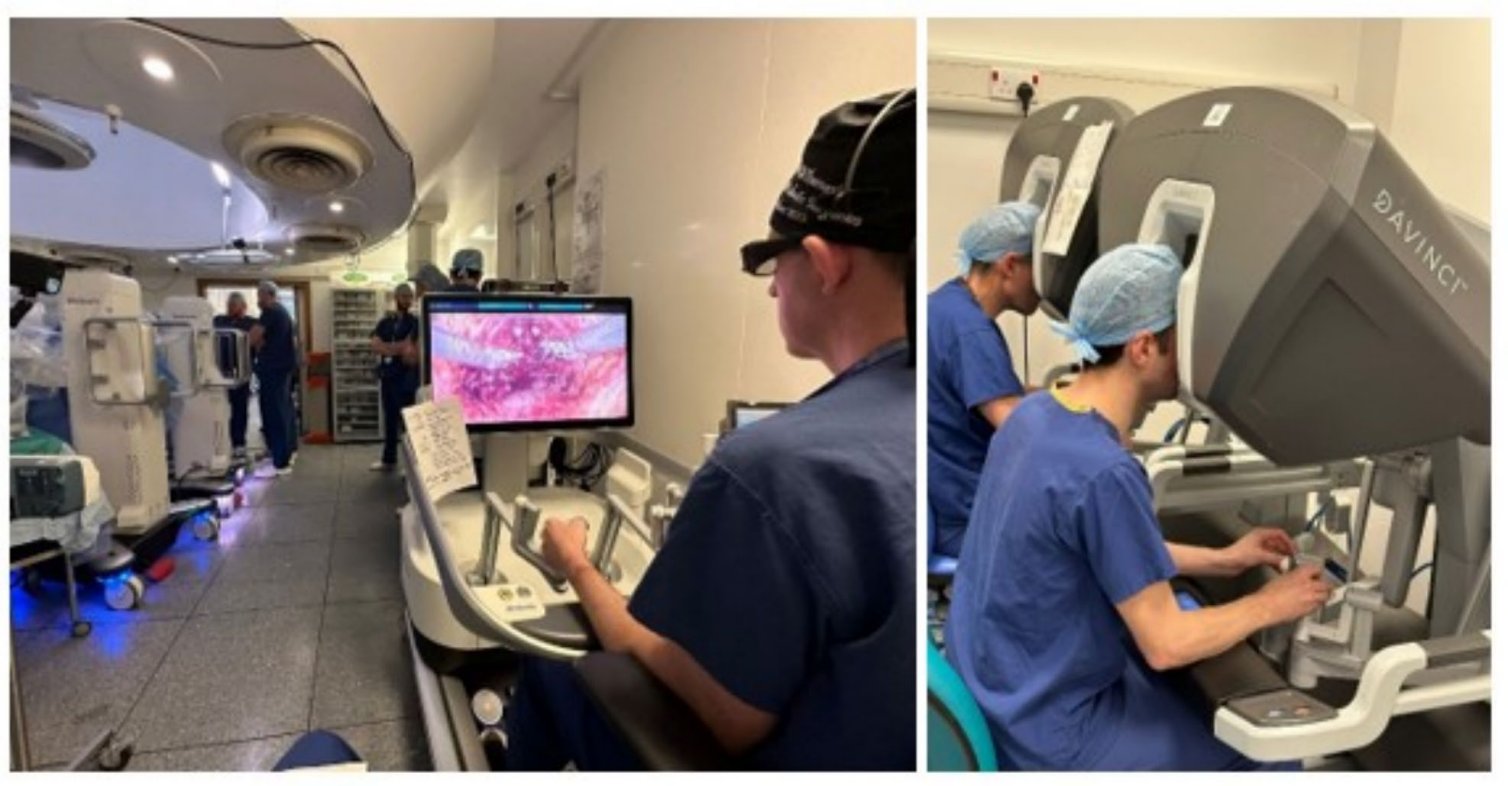
Comparison of console variations between the closed design of da Vinci^®^ (right) vs open Hugo^™^ (left)

**Fig. 2:**
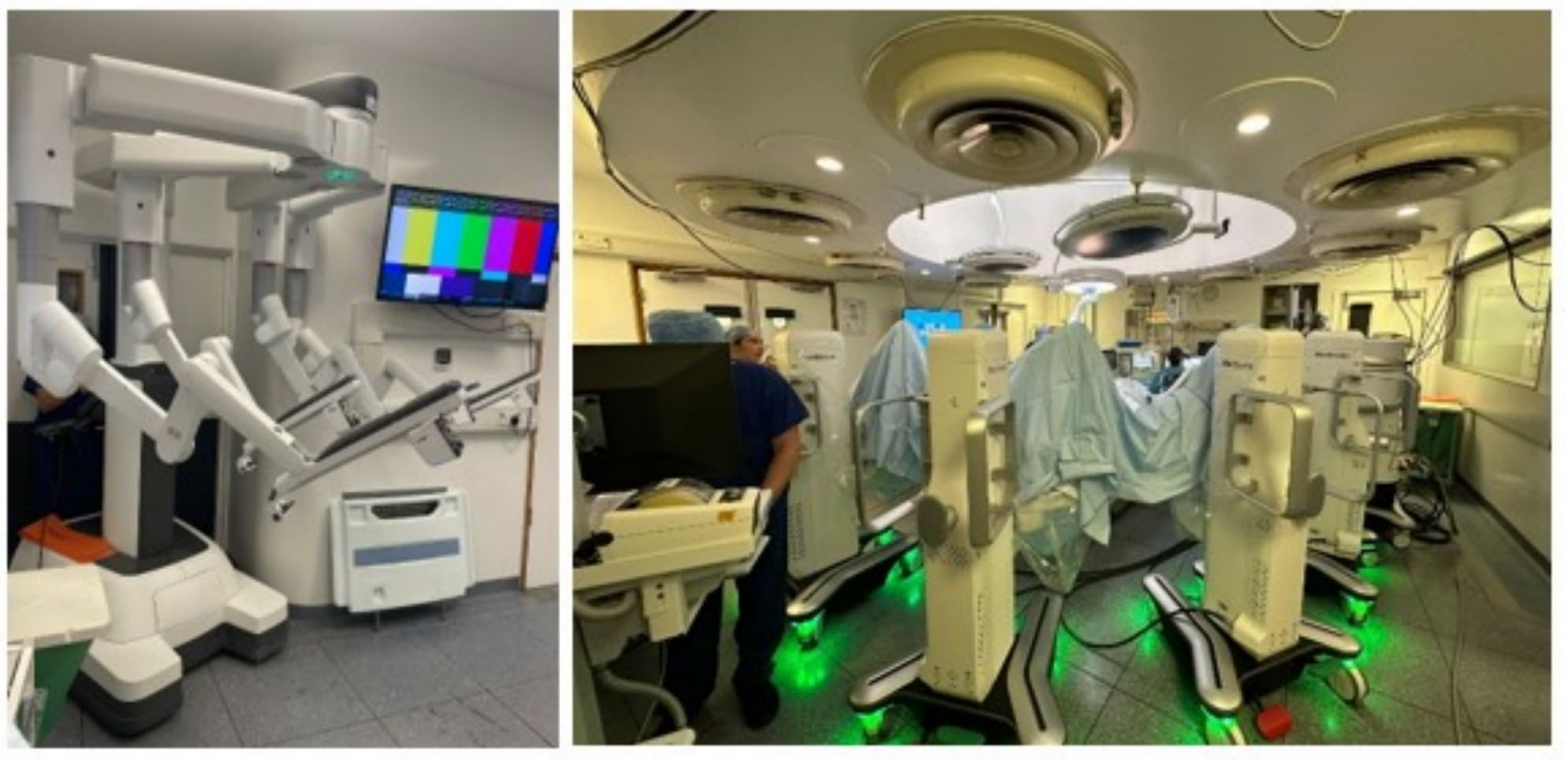
Comparison of patient arm cart setup in da Vinci^®^ (left) vs Hugo^™^ (right), displaying modularity of Hugo^™^ RAS

Medtronic's Hugo^™^ Robotic-Assisted Surgery (RAS) system stands out as a particularly promising alternative, displaying advanced robotic technology, artificial intelligence, and cutting-edge imaging capabilities [5] [6]. Notably, it enhances the existing robotic platforms by introducing an open console, fostering seamless communication amongst the surgical team [5]. Additionally, the system features a modular set of patient arm carts, amplifying versatility in surgical approaches. These comparisons can be viewed in Figs. 1 and 2, respectively.

Robotic-Assisted Radical Prostatectomies (RARP) took over from laparoscopic techniques in the early 2000s at centres capable of implementing the new robotic platforms [7]. This transition was credited to the successful mitigation of laparoscopic limitations and downsides to open surgery, with enhancements in operative, functional, and oncological outcomes [8].

Currently, reported cases at centres using the Hugo^™^ RAS are still limited due to the novelty, but with more regulatory boards granting approval for its use, the numbers are growing. The team at Guy’s and St Thomas’ NHS Trust, a well-established high-volume robotic surgery centre, is conducting an ongoing comparative study using the IDEAL framework to evaluate the Hugo^™^ RAS system in urology. Preliminary findings suggest that this system offers peri-operative and oncological outcomes comparable to those of the da Vinci^®^ system for RARP, indicating that Hugo^™^ RAS is a safe and viable alternative [9]. As surgical teams gain experience on the platform and confidence grows, its implementation in a wider array of procedures will follow.

RARPs are the most commonly conducted procedure with robotic assistance within urology [10], and therefore, a comprehensive understanding of outcomes and morbidities of this procedure has been well established. This creates practical areas for analysis of new systems to ensure comparable efficacy and safety. Data suggesting equivalence between platforms as a minimum can lead to the advantages brought by updated systems to optimise procedures. Greater affordability of Medtronic’s Hugo^™^ RAS could improve access to robotics at many centres.

With the Hugo^™^ RAS platform becoming integrated into an expanding number of centres worldwide for use in urology and particularly RARP procedures, we set out to systematically review experiences of this new system, comparing its safety and feasibility to other clinically available robotic systems.

## Methods

This study used the preferred reporting items for systematic reviews and meta-analyses (PRISMA) guidelines for protocol creation, and was registered on the Prospective Register of Systematic reviews [11] with the reference: CRD42024504844.

We used a comprehensive search strategy to identify relevant articles comparing the safety and efficacy of other robotic surgical systems for prostate cancer (Appendix 1). Population was defined as patients aged 18 years or older with prostate cancer (clinical stage T1 to T3, N0, M0), who underwent RARP; intervention was the Medtronic Hugo^™^ RAS system; for comparator, where possible or published, Hugo^™^ data compared with the existing robotic surgical outcome data using other robotic-assisted surgical systems and outcomes operatively, functionally, and oncologically. Following a scoping search, terms were adapted to include relevant synonyms. The following databases were used to carry out the search: MEDLINE, EMBASE, Cochrane Central Register of Controlled Trials, Scopus, Web of Science, clinicaltrials.gov, and World Health Organization (WHO) international clinical trials registry platform (ICTRP). Manual forward and backward citation searching was conducted for eligible studies, as well as hand searching of the five most cited urological/robotic journals (European Urology, BJUI, World Journal of Urology, The International Journal of Medical Robotics and Computer assisted Surgery, and Journal of Endourology [2018–2024]). Studies were collated using Endnote Clarivate, where duplicates were removed. The initial search was conducted on 22/01/2024, with a following top-up search carried out on 09/09/2024 finding additional articles included.

**Fig. 3:**
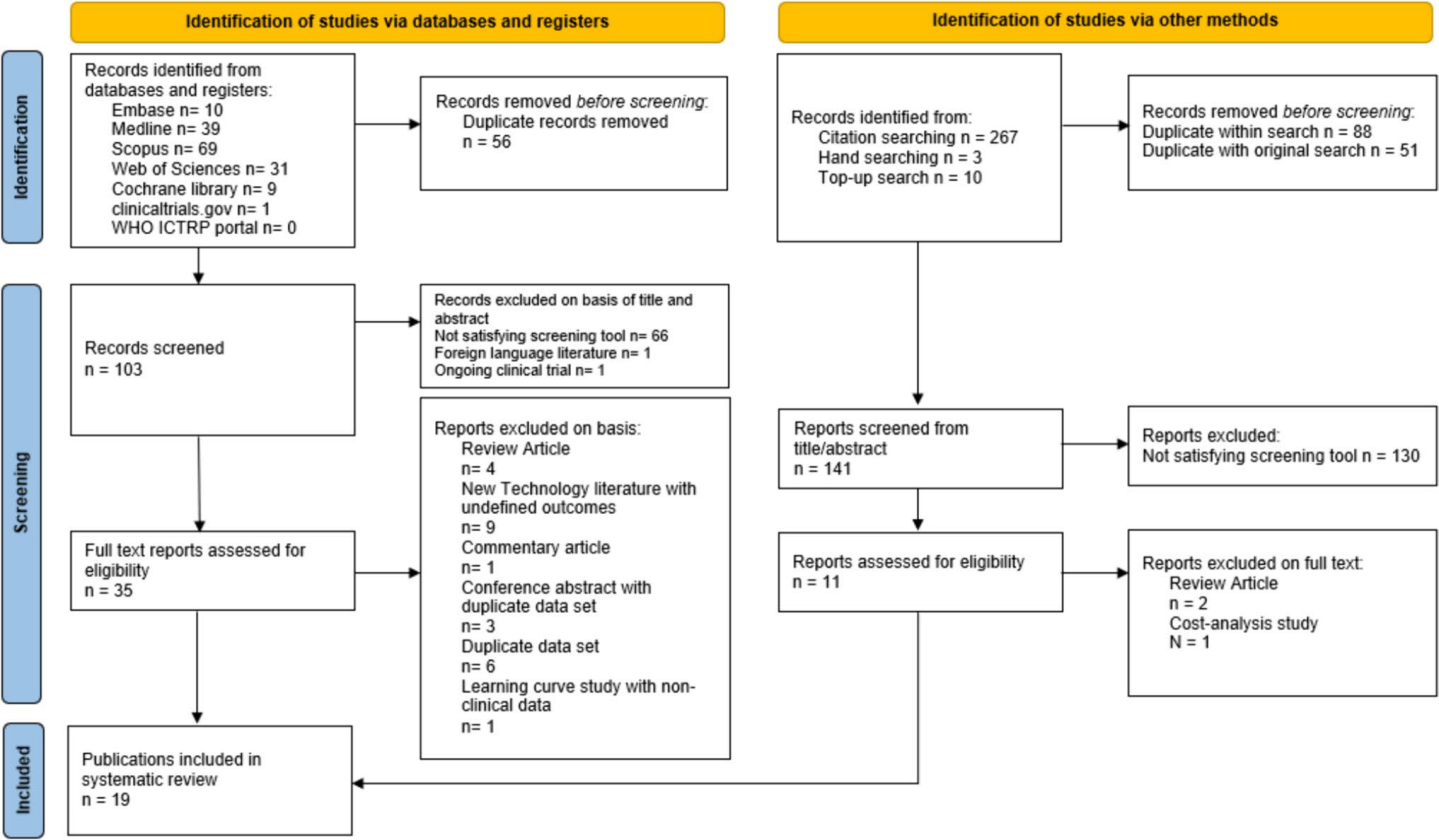
PRISMA flow diagram summary of the literature search and systematic review process

Two authors independently conducted each stage of screening (MST, AS), with any differences resolved in consensus meetings, or through consulting a third reviewer, (BC) if needed. An initial screening tool was applied to the titles and abstracts to include studies with direct comparisons between data from robotic platforms or descriptive comparisons between Hugo^™^ RAS experiences and current practise (other robotic platforms, laparoscopy, or open surgery). We included case-series, cohort studies, case–control studies, randomised control trials, systematic reviews, and meta-analyses meeting our inclusion criteria. We then conducted full-text review of included papers according to our predetermined PICO criteria [12]. Furthermore, we did not exclude relevant studies that do not report outcomes of interest of this review; and rather, summarised the key findings of any such studies. Reasons for exclusion were recorded and can be viewed in Fig. 3. Whilst there were no limitations based on language, logistical constraints necessitated the retrieval of non-English papers only if they had an accessible English abstract or full-text translation.

The same reviewers (MST, AS) then extracted data based on study characteristics, participant characteristics, intervention characteristics and outcomes, as well as any study funding sources for analysis. Risk-of-bias assessment implemented the ROBINS-I (Risk of Bias in non-randomised Studies of Interventions) [13] approach to assess methodological quality, appraising the following domains: confounding, participant selection, intervention classification, intervention deviations, missing data, measurement of outcomes, and reporting of results. Data analysis for quantitative findings included risk ratios (RR) for dichotomous outcomes and mean differences (MD) for continuous outcomes, accompanied by 95% confidence intervals (CI). In the case of observational studies such as case–control studies, non-randomised trials and cohort studies, we intended to highlight effect estimates as adjusted RR or odds ratios (OR), with corresponding 95% CI. If such data were unavailable, we used unadjusted RR or with 95% CI, P values only, or percentages in tabular format.

## Results

Figure 3 documents the search results and exclusions conducted at various stages of screening. The initial search screening tool found 36 potentially relevant studies; with 14 assessed as eligible following full-text review. The top-up search then found an additional 8 studies. One of these was the full-text update to a conference abstract included initially (Brime Menendez et al. 2024 paper for 2023 abstract [14, 15]). A further two were studies from the same centre with more recent data thus replacing the initially included paper (Gandi et al. 2024 update over Totaro et al. 2024 and 2022 [16–18]).

**Fig. 4:**
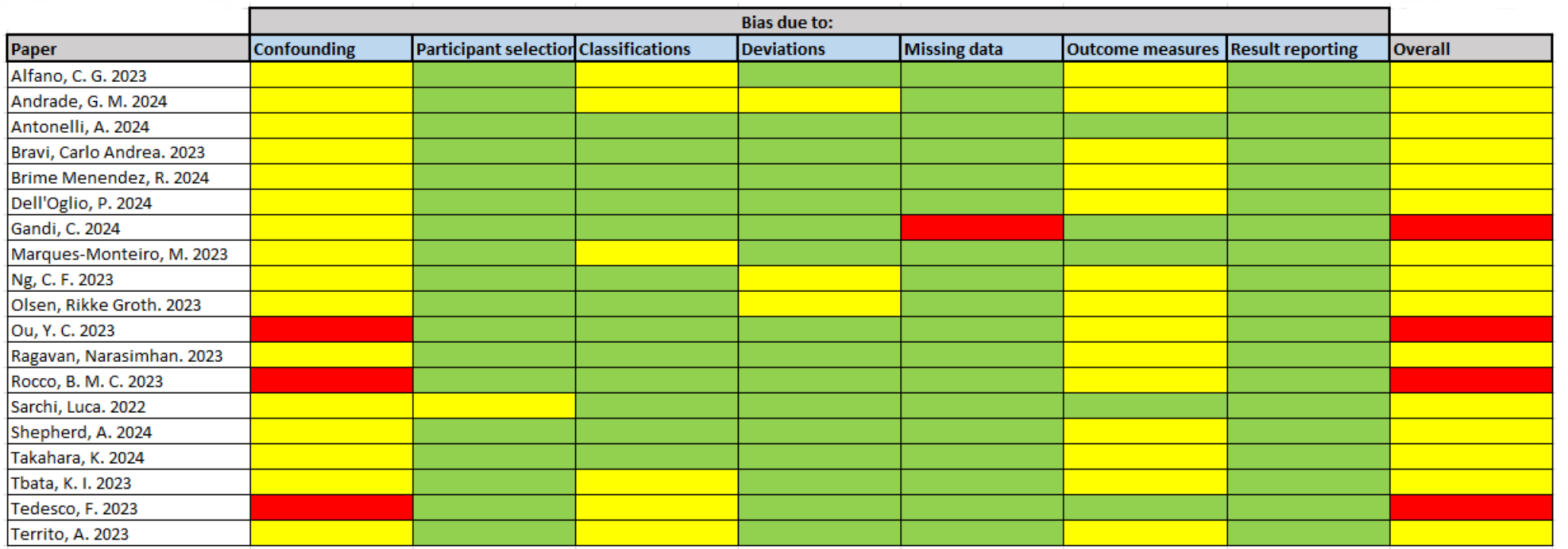
Risk-of-bias summary for each study

Table 1 presents an overview of the key characteristics of the included articles. Of the 19 included studies, 9 were comparative almost exclusively between Hugo^™^ and da Vinci^®^ with a single paper by Rocco et al. including the CMR Versius^™^ system. Notably, two of the studies lack quantitative values for their results, yet they offer valuable insights through narrative explanations. These studies, conducted by Rocco et al. (2023) [19] and Sarchi et al. (2022) [20], contribute diverse perspectives. Sarchi et al. conducted a cadaveric study, furnishing a comprehensive guide for the setup and docking of the novel Hugo^™^ robotic platform for RARP. Whilst the conference abstract by Rocco et al. conducted an evaluation focussing on safety aspects, analysing system errors across three platforms: da Vinci^®^, Hugo^™^, and Versius^™^. The study concluded that Hugo^™^ experienced three non-critical alarms and an instrument change, yet these events had no discernible adverse clinical or surgical impact (Fig. 4).

**Table 1:** Characteristics of eligible studies

Author	Country	Robotic platforms	Study type	Hugo^™^/da Vinci^®^ (n)	Main aim	Outcomes
Alfano, C. G. et al. 2023	USA	Hugo^™^	Retrospective cross-sectional	15	Operative outcomes	Acceptable peri-operative parameters and without intra-operative complications or conversions
Andrade, G. M. et al. 2024	Brazil	Hugo^™^	Retrospective cross-sectional	19	Operative and functional outcomes	Satisfactory peri-operative and functional parameters with safety and feasibility confirmed
Antonelli, A**.** et al. 2024	Italy	Hugo^™^ and da Vinci^®^	Prospective case–control	50/50	Evaluation of operative outcomes in early series	Longer operative time and more malfunctioning events in Hugo^™^ but comparable peri-operative outcomes
Bravi, Carlo Andrea. et al. 2023	Belgium	Hugo^™^ and da Vinci^®^	Retrospective case–control	164/378	Operative and functional outcomes in Hugo^™^ vs da Vinci^®^	No significant difference between platforms. Comparable peri- and post- operative as well as functional outcomes
Brime Menendez, R. et al. 2024	Spain	Hugo^™^ and da Vinci^®^	Prospective case–control	75/75	Operative and functional outcomes of Hugo^™^ compared with da Vinci^®^	Comparable operative and functional outcomes. Safety and feasibility confirmed for Hugo^™^ RARP
Dell’Oglio, P. et al. 2024	Italy	Hugo^™^	Retrospective cross-sectional	26	Operative and functional outcomes for Retzius-sparing RARP on Hugo^™^	Comparable peri-operative outcomes with promising functional outcomes. But non-negligible rate of positive surgical margins
Gandi, C. et al. 2024	Italy	Hugo^™^ and da Vinci^®^	Retrospective case–control	103/276	Operative and functional outcomes of Hugo^™^ compared with da Vinci^®^	Comparable operative and functional outcomes in propensity score matched group of da Vinci^®^. Safety and feasibility confirmed for Hugo^™^ RARP
Marques-Monteiro, M. et al. 2023	Portugal	Hugo^™^	Retrospective cross-sectional	16	Safety and docking setup	Extraperitoneal RARP approach safe and feasible with Hugo^™^. With uncomplicated transition from laparoscopy to robot-assisted
Ng, C. F. et al. 2023	China	Hugo^™^ and da Vinci^®^	Prospective case–control	10/20	Transfer of skills (learning curve + operative outcomes)	Comparable peri-operative outcomes with short learning curve for transition from da Vinci^*®*^ to Hugo^™^
Olsen, Rikke Groth. et al. 2023	Denmark	Hugo^™^ and da Vinci^®^	Prospective case–control	19/11	Transfer of skills (learning curve + operative outcomes)	Short learning curve for transition. Without compromise to operative outcomes. But no advantage to Hugo^™^ over da Vinci^®^
Ou, Y. C. et al. 2023	Taiwan	Hugo^™^	Prospective case-series	12	Transfer of skills (learning curve + operative outcomes)	Short learning curve based on console time for transition but with benefit from prior experience on da Vinci^®^
Ragavan, Narasimhan. et al. 2023	India	Hugo^™^ and da Vinci^®^	Prospective case–control	17/17	Operative outcomes of Hugo^™^ with matched da Vinci^®^ group	No significant difference between platforms. Comparable peri- and post- operative as well as functional outcomes
Rocco, B. M. C. et al. 2023	Italy	Hugo^™^, da Vinci^®^, and Versius	Prospective cross-sectional	1/1/1	Safety of Hugo^™^ and Versius against da Vinci^®^	Preliminary safety of Hugo™ with three non-critical errors requiring instrument change but no clinical/surgical impact
Sarchi, Luca. et al. 2022	Belgium	Hugo^™^	Preclinical case study	1	Preclinical safety study and docking setup	Confirmation of preclinical safety in cadaveric model. Plus, optimal docking setup
Shepherd, A. et al. 2024	UK	Hugo^™^ and da Vinci^®^	Prospective case–control	50/50	Operative and oncological outcomes of Hugo^™^ with matched da Vinci^®^ group	Initial learning curve for Hugo^™^ but peri-operative and oncological outcomes on par with da Vinci^®^ as well as equivocal safety
Takahara, K. et al. 2024	Japan	Hugo^™^	Retrospective cross-sectional	13	Operative outcomes and safety	Favourable peri-operative outcomes with safety and feasibility confirmed
Tbata, K. I. et al. 2023	Japan	Hugo^™^	Retrospective cross-sectional	10	Operative outcomes and safety	Comparable peri-operative outcomes without intra-operative conversions or complications
Tedesco, F. et al. 2023	Italy	Hugo^™^	Retrospective cross-sectional	30	Operative outcomes in 3-arm study	Confirmation of safety and feasibility of 3-asrms setup for RARP. Proving versatility
Territo, A. et al. 2023	Spain	Hugo^™^	Retrospective cross-sectional	20	Operative outcomes and safety	Acceptable peri-operative parameters and without intra-operative complications

**Fig. 5:**
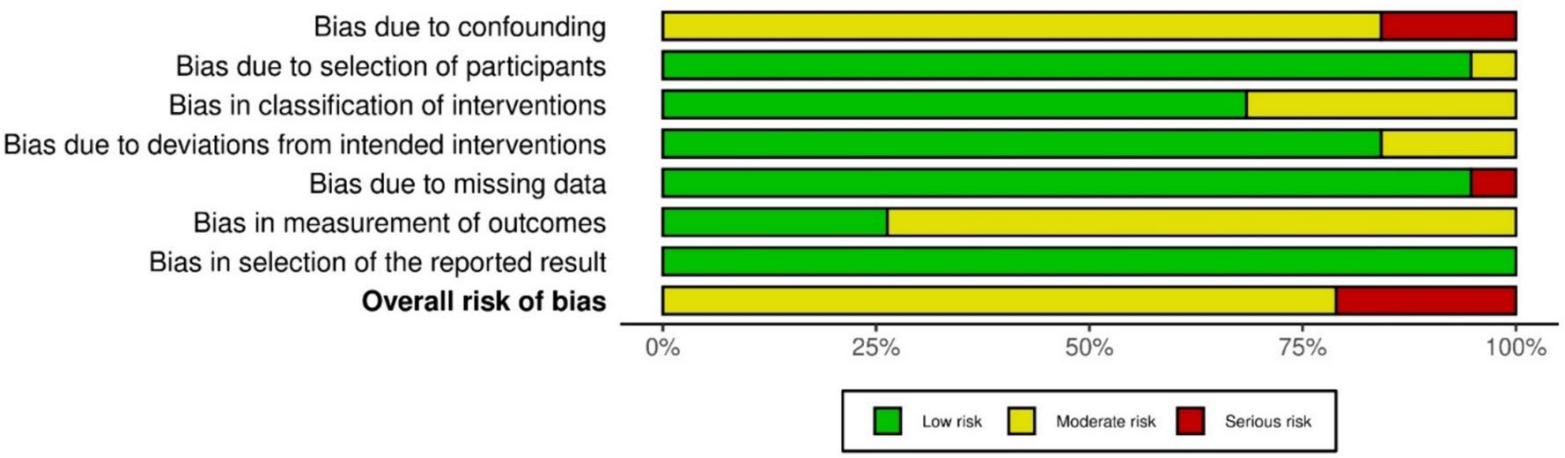
Bar chart illustration of risk-of-bias assessment in eligible studies

The quality of eligible studies was assessed using the ROBINS-I tool [13], showing serious risk of bias for four studies and moderate risk in remaining studies. Figure 5 demonstrates proportions of studies at each level of bias risk for the domains alongside the overall risk-of-bias performance. The main contributing domain to bias was confounding, attributable to a majority of eligible studies lacking randomisation. Bias in measurement outcomes was a factor for moderate risk in a high proportion of studies as assessors could not be blinded to the intervention and outcomes could be affected by their knowledge, such as in the case of a more cautious approach to the procedure in early cases of the implementation of a novel robotic system. One paper had serious risk in the domain for missing data due to the exclusion criteria of cases with conversions to open surgery. Also of note, the study by Antonelli, et al. was an open-label, non-randomised clinical trial [21].

Due to the high heterogeneity between the included studies, a wholly narrative synthesis was undertaken, forming a thematic analysis through the tabulation of the results of the studies for assignment into a framework of structured themes. This consisted of the following factors: operative time and its breakdown, safety of the Hugo^™^ RAS, participant demographics, and transfer of skills.

## Operative timings

The Hugo^™^ RAS employs a modular arm setup with individual carts to allow for greater versatility of docking setups based on various procedures, laterality, and location [22]. In addition to providing a more adaptable platform to optimise configurations based on surgeon or operating room requirements. This does, however, bring forth the issue of implementation within surgical teams experienced with the da Vinci^®^ platform. Patient positioning and trocar placements are mostly unchanged, but cart distances and angulation differ to achieve correct triangulation.

Studies found that the docking times for the initial cases exceeded da Vinci^®^ times greatly but did improve quickly [23]; 15 min down to 7 min as explained by Alfano et al. [24]. Although median times were still longer when compared to da Vinci^®^, with Olsen et al. [25] highlighting a statistically significant difference (*P* = 0.04) of 8 min with Hugo^™^ against 3 min. Total operative times are comparable between eligible studies for Hugo^™^, whilst console times contain more variation based on procedure approach and experience with robotic systems [26, 27]. Gandi et al. even show comparable total operative times between systems in a matched cohort, with Shepherd, et al. illustrating the same for console times [16, 27]. When compared with da Vinci^®^, conflict arises between studies with Bravi et al. finding significantly (*P* < 0.05) raised operative times for Hugo^™^ [28], yet Ragavan et al. concluding significantly (*P* < 0.05) reduced Hugo^™^ time [26]. Meanwhile, Olsen et al. determining no clinically relevant difference between systems [25]. Menendez et al. have produced the same conclusion, even with statistically significant reductions in the Hugo^™^ cohort when steps of the procedure are broken down [14]. This could possibly be attributable to confounders for instance patient disease stage and surgical team experience. Antonelli et al. remarks that the early longer timings can also be attributed to more meticulous care taken due to the novelty of the device [21]. Table 2 collates the findings of these 12 studies for operative timings in Hugo^™^ alongside any comparison. Current understanding explains on average longer operation times for Hugo^™^ in comparison to da Vinci^®^, due to challenges with docking setup of modular arm carts. However, with experience, this improves alongside shortening of isolated console times which becomes even faster than on da Vinci^®^.

**Table 2:** Summary of studies in operative timings theme

Study	Case number	Hugo^™^ timings (minutes, Median [IQR])	Comparable timings (minutes, Median [IQR])	Findings (significance at *P* < 0.05)
Alfano, C. G. et al. 2023	15 Hugo^™^	Total: 235 [213–271]	N/A	Docking process longer on Hugo^™^ and is the rate limiting step to reducing operative time with experience
**A**ndrade, G. M. et al. 2024	19 Hugo^™^	Console: 78 [60–120] (Range, not IQR)	N/A	Docking duration prolonged due to early inexperience
Bravi, Carlo Andrea. et al. 2023	164 Hugo^™^/378 da Vinci^®^	Total: 180 [150–200]	Total: 165 [130–200]	Significantly higher operative time on Hugo^™^ vs da Vinci^®^ particularly in pelvic lymphadenectomy cases
Brime, Menendez, R. et al. 2024	75 Hugo^™^/75 da Vinci^®^	Total: 139 [No IQR] (Mean not median)	Total: 145 [No IQR] (mean not median)	Non-significantly difference total operative times but significantly longer docking time for Hugo^™^. Breakdown of stages shows significant reductions for some in Hugo^™^
Dell’Oglio, P. et al. 2024	26 Hugo^™^	Total: 215 [181–262] Console: 156 [143–197]	N/A	Promising console times but with prolonged docking in early cases causing increased operative times
Gandi, C. et al. 2024	103 Hugo^™^/276 da Vinci^®^	Total: 170 [147–206]	Total: 170 [148–196]	No significant difference in total operative time between systems in unmatched and PSM matched cohorts
Olsen, Rikke Groth. et al. 2023	19 Hugo^™^/11 da Vinci^®^	Console: 97 [87–120]	Console: 89 [77–94]	Significantly longer docking, undocking, and bladder-neck dissection times on Hugo™ with non-significantly higher console time adjusted for lymph-node dissection
Ou, Y. C. et al. 2023	12 Hugo^™^	Console: 145 [130–170]	N/A	Shorter console times on early Hugo™ cases than early da Vinci^®^ cases with significant reductions for one surgeon over time
Ragavan, Narasimhan. et al. 2023	17 Hugo^™^/17 da Vinci^®^	Total: 195 [180–240] Console: 170 [160–205]	Total: 210 [210–240] Console: 190 [180–210]	Hugo™ has a significantly shorter operative time and non-significant reduction in console time vs. da Vinci^®^, with longer docking time improving after finding the ideal layout
Shepherd, A. et al. 2024	50 Hugo^™^/50 da Vinci^®^	Console: 148 [90 – 230)] (Mean [Range, not IQR])	Console: 149 [80–240] (Mean [Range, not IQR])	Non-significant difference in console times
Takahara, K. et al. 2024	13 Hugo^™^	Total: 197 [187–228] Console: 134 [125–157]	N/A	Acceptable console times but breakdown into stages of procedure gives too much variation to determine learning curve
Tedesco, F. et al. 2023	30 Hugo^™^	Console: 120 [100–150]	N/A	Acceptable operative times for three-arm setup of Hugo^™^

## Transfer of skills

One of the key components for transitioning to a novel robotic platform is the transfer of skills from previously used systems for both the surgeon and surgical team.

Eligible studies have highlighted that centres which will be implementing the Hugo^™^ RAS undergo adequate dry and wet lab training provided by Medtronic, vital in assisting the team with adapting to the new platform [24, 25]. Adoption of the new system designs, namely pistol-like hand controls and modular arms were the aspects requiring a longer learning curve in the studies. Nevertheless, surgical experience with a robotic platform has been shown to transfer to Hugo^™^ in a rapid period; Ng et al. and Ou et al. showing transitions under ten cases [23, 29]. Antonelli et al. present a comprehensive longitudinal analysis of procedure timings, concluding that due to the novelty of the Hugo^™^ system, both operative time and setup duration are initially longer [21]. However, with increased experience, these times improve, reflecting the expected learning curve associated with the device. Table 3 aggregates the five relevant studies assessing transfer of skills and learning curves for Hugo^™^; highlighting short curves for early adoption of the new system which is even faster for surgeons with prior robotic experience. Allowing for unhindered transfer from da Vinci^®^. Studies into robot-naïve surgeons will be the next step to understanding learning progress for the Hugo^™^.

**Table 3:** Summary of studies in transfer of skills theme

Study	Case number	Learning curve	Findings
Antonelli, A. et al. 2024	50 Hugo^™^/50 da Vinci^®^	Short	Based on longitudinal operative time analysis
Bravi, Carlo Andrea. et al. 2023	164 Hugo^™^/378 da Vinci^®^	Short	Based on operative timing. Initial phase of learning Not linked to surgical experience
Ng, C. F. et al. 2023	10 Hugo^™^/20 da Vinci^®^	Short	Skill transfer achieved in under 10 cases with comparable peri-operative outcomes and operative times
Olsen, Rikke Groth.et al. 2023	19 Hugo^™^/11 da Vinci^®^	Short	Peri- and post-operative parameters suggest very short learning curve for experienced robotic surgeons
Ou, Y. C.et al. 2023	12 Hugo^™^	Short	Quick learning curve with no peri-operative complications. Shorter curve with more robotic experience

## Participant demographics

Understanding the safety, feasibility, and operative outcomes from research into new robotic platforms requires the use of representative patient cohorts. Being selective with cases that can be performed by Hugo^™^ is important for maintaining patient safety, stemming from being unacquainted with its capabilities and limitations. Even so, selection criteria should be adjusted with increasing surgeon confidence to enable comparable patient demographics with previously used platforms. Olsen et al. found that patients for which Hugo^™^ was used had a larger BMI range and higher cancer staging [25]. The other comparative studies also lacked any statistically significant difference between their patient cohorts, indicating a level of reliability to their findings [14, 16, 21, 29]. Overall, conveying the unrestrictive nature of patient selection and range in case complexities for the Hugo^™^ cohorts. Therefore, the comparisons made with da Vinci^®^ are between similar oncological and patient specific demographics, ensuring robust internal validity.

## Safety and feasibility

In order for a recently launched intervention, particularly a robotic platform, to gain widespread acceptance from surgeons and their respective centres, it has to successfully show safety in its early cases that parallels the precursor. In the instance of Hugo^™^, rates of complications and positive surgical margins are a key variable to consider as comparable to da Vinci^®^. Following data synthesis, it is evident that although complications (Clavien–Dindo ≥ 2) may arise from either system; it needs to be differentiated whether the device was responsible/linked or an inherent risk associated with the procedure itself, such as wound closure was the cause. Occurrences of conversion to laparoscopic, alternate robotic platforms, or open is another crucial aspect of safety assessment.

Olsen et al. demonstrated that an experienced robotic surgeon can successfully switch from da Vinci^®^ to Hugo^™^ without a clinically relevant dip in performance; although no improvements were observed for operative outcomes, there were also no compromises due to complications [25]. The complications that did arise were due to quality of the closure and had been previously described for da Vinci^®^ [30]. This study also showed that positive surgical margin rates were comparable between the systems, even though marginally higher in Hugo^™^. Bravi et al., Shepherd et al., and Gandi et al. also recognised no statistically significant difference between the platforms for complications and positive margin rates; however, an issue with the latter is an exclusion criteria for cases converted to open [16, 27, 28]. Moreover, Dell’Oglio et al. found non-negligible rates of positive surgical margins when comparing with other related papers. Alfano et al. illustrated safety with the use of Hugo^™^ at their centre owed to a lack of intra-operative complications, a single non-robotic post-operative complication, and positive margin rates in accordance with the other studies. This result was supported by Ou et al., Marques-Monteiro et al., and Territo et al. alongside the latter two studies indicating no mechanical failures to have emerged [31]. With regard to malfunctioning and technological issues, four studies directly concluded that mechanical failures were absent. These were the two mentioned prior as well as Sarchi et al. and Takahara et al., whilst other included papers made no mention of such faults. Andrade et al. also mention the recurring issue with arm collisions [32, 33]. The presence of arm and monitor failures causing disruptions to operative flow have been reported, however, with Antonelli et al. presenting a multitude of malfunctioning/troubleshooting events that did not lead to conversion or complication [17, 21]. These are to be expected as Hugo^™^ RAS is currently at its first edition; therefore, recognition of these failures are key to improving safety. Bringing these to light will ensure that software issues are resolved through scheduled updates and more major mechanical compromises are acted on to prevent, with failures functioning as learning points for future generations of the device. March 2024 saw the implementation of a significant software update that solved prior issues with alarms and arm clash errors. This demonstrates how Medtronic is actively listening to feedback from Hugo RAS^™^ users to implement changes that address faults and enhance the system.

Table 4 summarises the findings of 17 studies evaluating the safety and feasibility of Hugo^™^. It presents peri-operative outcomes, including estimated blood loss, positive surgical margins, and the presence of intra-operative complications or conversions. These metrics, used by the majority of eligible studies, highlights the efficacy and feasibility of Hugo^™^ as an alternative to da Vinci^®^. Furthermore, no eligible studies mention instances of conversions to open, laparoscopy or another robotic platform. Another domain to consider is also the functional outcomes, particularly continence and erectile function. Data on these variables are not always readily available in the literature due to the novelty of the research. However, seven of the included studies do incorporate it with early results being encouraging. Two studies delved into erectile function through use of IIEF-5 scoring and found comparability [14, 21]. Urinary continence recovery at 1 and/or 3 months were found to be acceptable and in most cases comparable, only 1 study concluded significant difference in favour of da Vinci^®^ [14, 24–26, 28]. With current research demonstrating an absence of serious peri-operative complications as well as any post-operative complications unrelated to the robotic system, there is a promising outlook for the safety of Hugo^™^.

**Table 4:** Summary of studies in safety and feasibility theme

Study	Case number	EBL (mL, median [IQR])	Positive surgical margins (n [%])	Conversions or intra-operative complications
Alfano, E. G. et al. 2023	15 Hugo^™^	300 [100–310]	5 [33%]	None
Andrade, G. M. et al. 2024	19 Hugo^™^	“ < 200 in all patients”	1 [5%]	None
Antonelli, A. et al. 2024	50 Hugo^™^/50 da Vinci^®^	Hugo^™^: 300 [150–400] Da Vinci^®^: 200 [150 –300]	Not stated	None
Bravi, Carlo Andrea. et al. 2023	164 Hugo^™^/378 da Vinci^®^	Hugo^™^: 400 [250–500] Da Vinci^®^: 350 [200–500]	Hugo^™^: 20 [12%] Da Vinci^®^: 55 [15%]	Not stated
Brime**,** Menendez, R. et al. 2024	75 Hugo^™^/75 da Vinci^®^	Hugo^™^: 250 [No IQR] (Mean not median) Da Vinci^®^: 330 [No IQR] (Mean not median)	Hugo^™^: 15 [20%] Da Vinci^®^: 8 [11%]	None
Dell’Oglio, P. et al. 2024	26 Hugo^™^	90 [54–100]	8 [31%]	None
Gandi, C. et al. 2024	103 Hugo^™^/276 da Vinci^®^	Hugo^™^: 100 [100–150] Da Vinci^®^: 100 [100–155]	Hugo^™^: 23[22%] Da Vinci^®^: 58 [21%]	None
Marques-Monteiro, M. et al. 2023	16 Hugo^™^	200 [150–400]	Not stated	None
Ng, C. F. et al. 2023	10 Hugo^™^/20 da Vinci^®^	Hugo^™^: 200 [200–500] Da Vinci^®^: 350 [300–525]	Not stated	Not stated
Olsen, Rikke Groth. et al. 2023	19 Hugo^™^/11 da Vinci^®^	Hugo^™^: 300 [150–400] Da Vinci^®^: 200 [100–350]	Hugo^™^: 7 [37%] Da Vinci^®^: 3 [27%]	None
Ou, Y. C. et al. 2023	12 Hugo^™^	193 [IQR = 226]	3 [25%]	None
Ragavan, Narasimhan. et al. 2023	17 Hugo^™^/17 da Vinci^®^	Not Stated	Hugo^™^: 4 [24%] Da Vinci^®^: 4 [24%]	None
Shepherd, A. et al. 2024	50 Hugo^™^/50 da Vinci^®^	Hugo^™^: 168 [50–650] (Mean [Range not IQR]) Da Vinci^®^: 208 [30–600] (Mean [Range not IQR])	Hugo^™^: 3 [6%] Da Vinci^®^: 8 [16%]	None
Takahara, K. et al. 2024	13 Hugo^™^	150 [80–250]	1 [8%]	None
Tbata, K. I. et al. 2023	10 Hugo^™^	135 [5–330] (Range not IQR)	1 [11%]	None
Tedesco, F. et al. 2023	30 Hugo^™^	150 [100–250]	Not stated	Not stated
Territo, A. et al. 2023	20 Hugo^™^	200 [150–250]	5 [29%]	None

## Discussion

Current literature delving into varying aspects of the new Hugo^™^ RAS platform by Medtronic (for RARP in prostate cancer patients) has a common agreement on the safety and applicability of the system. This is evidenced by a multitude of factors such as the short learning curves for transfer of skills from other robotic platforms or in robot-naïve surgeons, absence of intra-operative complications or conversions, comparable peri-operative outcomes, and competitive console times [23–26, 28, 29, 34, 35]. Experiences at multiple centres and with different surgeons have been positive in showing minimal change between this novel platform and the pre-existing conventional system, da Vinci^®^. This creates a foundation for more centres worldwide to involve the Hugo^™^ platform in their cases, widening understanding of the advantages it may bring to patient outcomes and safety, whilst learning of the shortcomings it may possess due to the limited experience Medtronic have in the robotic market. Concurring improvements to the platform, adaptations for a greater range of uses and developing competition in the market to push companies to deliver better products are positive implications. Inevitably enabling surgeons to have a system that suits their needs to bring refinements to patient care with a rivalling cost-effectiveness that centres can make the most of. Cost analysis comparing the Hugo^™^ and da Vinci^®^ has revealed a saving of 11% in total financial burden with the Hugo^™^ [36]. This cost advantage makes Hugo^™^ a more affordable option, which could significantly lower the barrier for many centres looking to adopt robotic surgery.

Another central finding of the review was the lack of high-quality evidence surrounding the topic as the system is new and no high-quality randomised trials have yet been attempted. Although the available literature points towards Hugo^™^ being a safe and appropriate alternative to da Vinci^®^ with comparable outcomes, the evidence base consists of observational studies as a majority. Following risk-of-bias assessment, a high proportion of studies were graded as moderate-to-serious risk of bias as a result of performance and detection bias from a lack of blinding, as well as selection bias from an absence of randomisation or unclear allocation criteria. Furthermore, much of the available articles contain qualitative accounts of their experience, and between those that are quantitative, there is much heterogeneity of measured parameters, preventing reliable pooled analyses. This study’s limitation stems from the scarcity of high-quality randomised-controlled trials, which impedes complete confidence in the conclusions able to be drawn. These, however, are inherent weaknesses from Hugo^™^ still being in the early stages of implementation with a growing number of centres using the platform. Further larger multicentre studies supporting these early results are the next stage in the evaluation and implementation of this new technology, following the IDEAL principles of evaluation [37]. To our knowledge, there is currently a single study with pooled statistical analysis of the comparison between Hugo^™^ and da Vinci^®^ [38]. The conclusions from this study ameliorate our findings, demonstrating the wide range of heterogeneity within the literature as well as comparable surgical, oncological, and functional outcomes. Thus, underscoring the safety, feasibility, and efficacy at this early stage with the necessity of more complete data. A key distinction of our paper, in contrast to their study, lies in the more comprehensive scope of our analysis, as we incorporate 19 articles instead of their 12.

## Conclusions

In conclusion, this systematic review assessed the safety and feasibility of the Medtronic Hugo^™^ Robotic-Assisted Surgery (RAS) system for Robotic-Assisted Radical Prostatectomy (RARP) in comparison to other robotic surgical systems, particularly the da Vinci^®^. The study examines operative timings, transfer of skills, participant demographics, safety, and oncological outcomes. Although limited by a scarcity of high-quality randomised-controlled trials, the current evidence suggests that Hugo^™^ is a safe alternative with comparable outcomes. The findings encourage wider adoption, anticipating refinements and cost-effectiveness, whilst highlighting the need for rigorous research to strengthen the evidence base for the evolving Hugo^™^ platform in other urological procedures or specialties.

## Figures and Tables

**Fig. 6:**
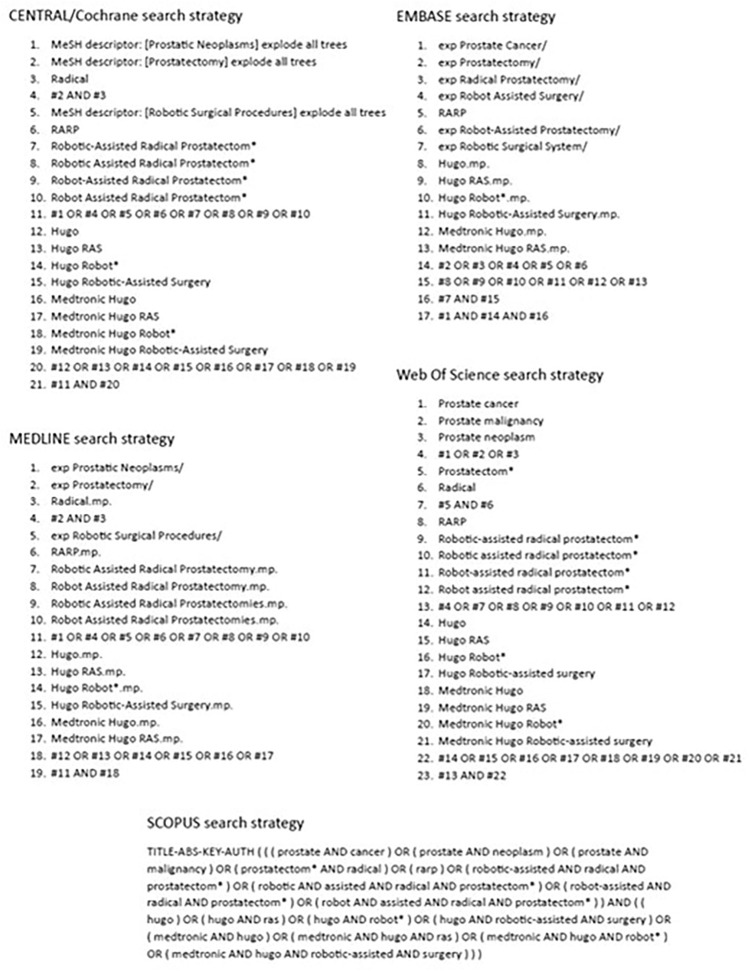
Search strategy applied for each database used

## Data Availability

No datasets were generated or analysed during the current study.

## Appendix 1

See Fig. 6.
